# Supplementary material for: Methicillin-Resistant Staphylococcus aureus Clonal Complex 398 as a Major MRSA Lineage in Dogs and Cats in Thailand
Source: Antibiotics (Basel). 2021 Feb 28;10(3):243. doi: 10.3390/antibiotics10030243 (PMC7997496; doi:10.3390/antibiotics10030243)
Supplement: Supplementary file 1 [file antibiotics-10-00243-s001.pdf]

antibiotics

Supplementary

Article

Methicillin-Resistant *Staphylococcus aureus* Clonal Complex 398 as a Major MRSA Lineage in Dogs and Cats in Thailand

Surawit Chueahiran [1], Jitrapa Yindee [1], Pongthai Boonkham [2], Nipattra Suanpairintr [3] and Pattrarat Chanchaithong [1,4,*]

1. Department of Veterinary Microbiology, Faculty of Veterinary Science, Chulalongkorn University, Bangkok 10330, Thailand; 6271026031@student.chula.ac.th (S.C.); jitrapa.y@chula.ac.th (J.Y.)
2. Veterinary Diagnostic Laboratory, Faculty of Veterinary Science, Chulalongkorn University, Bangkok 10330, Thailand; pongthat.b@chula.ac.th
3. Department of Pharmacology, Faculty of Veterinary Science, Chulalongkorn University, Bangkok 10330, Thailand; nipattra.d@chula.ac.th
4. Research Unit in Microbial Food Safety and Antimicrobial Resistance, Faculty of Veterinary Science, Chulalongkorn University Bangkok 10330, Thailand

* Correspondence: pattrarat.c@chula.ac.th

Table S1: Oligonucleotide primers for antimicrobial resistance gene detection.

Gene	Primer	Nucleotide sequence (5′→3′)	Annealing temperature (°C)	Extension time (second)	Amplicon size (bp)	Reference
blaZ	blaZ-F	CAGTTCACATGCCAAAGAG	50	45	772	Schnellmann et al., 2006. J Clin Microbiol. 44(12): 4444-4464
	blaZ-R	TACACTCTTGGCGGTTTC				
mecA	mecA-1	AAAATCGATGGTAAAGGTTGGC	55	30	532	Strommenger et al., 2003. J Clin Microbiol. 41(9): 4089-4094
	mecA-2	AGTTCTGCAGTACCGGATTTGC				
tet(K)	tet(K)-1	TTAGGTGAAGGGTTAGGTCC	54	45	697	Aaresterup et al., 2000. Diagn Microbiol Infect Dis. 37: 127–137.
	tet(K)-2	GCAAACTCATTCCAGAAGCA				
tet(L)	tet(L)-1	CATTTGGTCTTATTGGATCG	50	30	456	Aaresterup et al., 2000. Diagn Microbiol Infect. 37: 127–137.
	tet(L)-2	ATTACACTTCCGATTTCGG				
tet(M)	tet(M)-1	GTTAAATAGTGTTCTTGGAG	50	40	576	Aaresterup et al., 2000. Diagn Microbiol Infect. 37: 127–137.
	tet(M)-2	CTAAGATATGGCTCTAACAA				
aac(6′)-Ie-aph(2′)-Ia	aacA-aphD-1	CCAAGAGCAATAAGGGCATA	54	45	220	Van de Klundert and Vliegenthart, 1993. In: Diagnostic Molecular Microbiology. pp 547-552.
	aacA-aphD-2	CACTATCATAACCACTACCG				
ant(4′)-Ia	aadD-1	GCAAGGACCGACAACATTTC	54	30	165	Van de Klundert and Vliegenthart, 1993. In: Diagnostic Molecular Microbiology. pp 547-552.
	aadD-2	TGGCACAGATGGTCATAACC				
ant(6)-Ia	aadE-pS1	GCAGAACAGGATGAACGTATTCG	58	30	373	This study from GenBank nucleotide accession No. KF421157.1
	aadE-R	CTATATCAGTCGGAACTATGTCCC				
spw	spw-fw	CGGCAGTAATGGGTGGTTTA	54	45	630	

	spw-rv	CAGCCACCTCAGATTCCATT				Wendlandt et al., 2013. J Antimicrob Chemother. 68: 1679-1690.
erm(A)	erm(A)-F	TCTAAAGCATGTAAAGAA	48	60	645	Sutcliff et al., 1996. Antimicrob Agents Chemother. 40:2562–2566.
	erm(A)-R	CTTCGATAGTTTATTAATATTAGT				
erm(B)	erm(B)-F	GAAAAGGTACTCAACCAAATA	52	60	639	Sutcliff et al., 1996. Antimicrob Agents Chemother. 40:2562–2566.
	erm(B)-R	AGTAACGGTACTTAAATT-GTTTAC				
erm(C)	erm(C)-F	TCAAAACATAATATAGATAAA	43	60	642	Sutcliff et al., 1996. Antimicrob Agents Chemother. 40:2562–2566.
	erm(C)-R	GCTAATATTTAAATCGTCAAT				
lnu(B)	lnuB-F	CCTACCTATTGTTTGTGGAA	51	60	925	Bozdogan et al., 1999. Antimicrob Agents Chemother. 43(4): 925-929.
	lnuB-R	ATAACGTTACTCTCCTATTC				
lsa(E)	lsaE-F	ACGGACGCGGTAAAACTACT	54	45	693	Chanchaithong et al., 2014. J Appl Microbiol. 117: 572-586.
	lsaE-R	TTGGCACGTTTCATCGCTTT				
cat_{pC221}	catpC221-F	ATTTATGCAATTATGGAAGTTG	53	30	435	Schoenfelder et al., 2017. Vet Microbiol. 200: 79-87
	catpC221-R	TGAAGCATGGTAACCATCAC				
fexA	fexA-fw	GTACTTGTAGGTGCAATTAC-GGCTGA	61	90	1,272	Kehrenberg and Schwarz, 2006. Antimicrob Agents Chemother. 50(4): 1156-1163.
	fexA-rv	CGCATCTGAGTAG-GACATAGCGTC				
dfrA	dfrA-F	CCTTGGCACTTACCAAATG	50	30	374	Schnellmann et al., 2006. J Clin Microbiol. 44(12): 4444-4464
	dfrA-R	CTGAAGATTCGACTTCCC				
dfrG	dfrG-1	TCGGAAGAGCCTTAC-CTGACAGAA	58	30	323	Gómez-Sanz et al., 2010. Foodborne Path Dis. 7(1): 1269-1277.
	dfrG-2	CCCTTTTTGGGCAAATAC-CTCATTCCA				
ileS2	mupA	TATATTATGCGATGGAAGGTTGG	57	30	458	Anthony et al., 1999. J Clin Microbiol Infect. 18: 30-34
	mupB	AATAAAATCAGCTG-GAAAGTGTTG				
